# A Smart Healthcare Knowledge Service Framework for Hierarchical Medical Treatment System

**DOI:** 10.3390/healthcare10010032

**Published:** 2021-12-24

**Authors:** Yi Xie, Dongxiao Gu, Xiaoyu Wang, Xuejie Yang, Wang Zhao, Aida K. Khakimova, Hu Liu

**Affiliations:** 1 The School of Management, Hefei University of Technology, Hefei 230009, China; yixie928@163.com (Y.X.); 2019110768@mail.hfut.edu.cn (W.Z.); lu18752099127@163.com (H.L.); 2 The School of Environment, Education and Development, University of Manchester, Manchester M13 9PL, UK; 3 The Department of Pharmacy, Anhui University of Traditional Chinese Medicine, Hefei 230009, China; xywang0551@163.com; 4 Scientific-Research Center for Physical-Technical Informatics, Russian New University, 105005 Moscow, Russia; aida_khatif@mail.ru

**Keywords:** smart healthcare management, hierarchical medical treatment system, knowledge service, case-based reasoning

## Abstract

This paper reveals the research hotspots and development directions of case-based reasoning in the field of health care, and proposes the framework and key technologies of medical knowledge service systems based on case-based reasoning (CBR) in the big data environment. The 2124 articles on medical CBR in the Web of Science were visualized and analyzed using a bibliometrics method, and a CBR-based knowledge service system framework was constructed in the medical Internet of all people, things and data resources environment. An intelligent construction method for the clinical medical case base and the gray case knowledge reasoning model were proposed. A cloud-edge collaboration knowledge service system was developed and applied in a pilot project. Compared with other diagnostic tools, the system provides case-based explanations for its predicted results, making it easier for physicians to understand and accept, so that they can make better decisions. The results show that the system has good interpretability, has better acceptance than the common intelligent decision support system, and strongly supports physician auxiliary diagnosis and treatment as well as clinical teaching.

## 1. Introduction

According to a report of the World Health Organization in 2019, the world’s population aged over 60 years has exceeded 1 billion [1]. The number of patients with chronic diseases, such as cardiovascular diseases, cancer, diabetes, and chronic respiratory diseases, has increased rapidly, and the number of deaths caused by these diseases accounts for 74% of the world’s deaths [2]. According to the census data released by the National Bureau of Statistics, 260 million people are over 60 in China, accounting for 5.44% of the total population, and the mortality rate from chronic diseases accounts for 88.5% of the total mortality rate [3,4]. With the continuous improvement of people’s living standards, society’s demand for safe and high-quality medical and health services is growing rapidly. However, problems such as an insufficient amount of social medical resources, unbalanced allocation, low levels of primary medical services, and difficulties with the decreasing quality of medical resources still exist, all of which lead to difficulty in implementing a national hierarchical medical treatment system in developing countries like China. In many remote and underdeveloped areas, a big gap exists between the medical resources available, especially the level of medical skills, compared with developed areas. Therefore, high-quality medical services are unavailable for many patients. As a result, many patients choose to go to large hospitals that are far away from their homes, resulting in the phenomenon of “few patients in small hospitals and many patients in large hospitals”.

The arrival of the big data era has brought great challenges and opportunities for the development of medical health. On the one hand, the explosive growth of medical information resources presents new characteristics of being large scale, multimodal, multisource, heterogeneous, and dynamic, and traditional information technology cannot effectively process and use these complex data [5]. On the other hand, the rise of cloud computing, big data, artificial intelligence, and other aspects of the new generation of information technology enables the knowledge service system to effectively collect, store, process, and organize massive amounts of multisource and heterogeneous medical and health data, which helps improve the efficiency of disease diagnosis and the effectiveness of medical care [6,7]. Medical and health data shared across domains and organizations can comprehensively and deeply reflect the health status of residents, and this sharing shows great potential in pathogenesis, diagnosis, prevention, treatment, and prognosis [8,9]. For example, the Smart Asthma Management System (SAM) collects patient breathing data through Bluetooth-enabled inhalers, allows physicians to keep abreast of patient health and develop personalized treatment options, and allows patients to manage their own health through its systems [10]. Some artificial intelligence software can be used for data mining and knowledge discovery on the basis of electronic health records (EHRs) to provide decision support for physicians in clinical diagnosis and treatment [11]. Despite agreement about the importance of clinical big data in increasing the effectiveness and efficiency of medical care services, research on a healthcare knowledge service system that integrates general medical knowledge, clinical health data, and clinical bases is lacking. 

An approach to applying to the healthcare knowledge service is the use of case-based reasoning (CBR). The basic principle of CBR is to obtain similar past cases in accordance with the characteristics of current problems and then learn and solve these problems [12]. The medical decision-making process itself is highly dependent on the physician’s historical experience and knowledge. In essence, the principle of CBR is consistent with the physician’s manner of thinking. Therefore, it more easily accepted by physicians. Moreover, through the establishment of a case base, physicians could obtain knowledge as a decision-making reference to help improve their level of diagnosis and treatment [13,14,15]. CBR technology helps physicians in underdeveloped and remote areas to obtain information on the high-quality cases of large hospitals through the Internet, obtain valuable information through case studies, and improve the quality of diagnosis and treatment [16]. A CBR knowledge service system can also integrate general medical knowledge and clinical case knowledge and provide rich clinical expertise for primary and young physicians to assist with diagnosis and treatment and clinical thinking training [11]. This could provide convenience for patients and save time and economic costs [17]. Various CBR systems providing knowledge services to medical diagnosis, such as early detection of breast cancer, auxiliary diagnosis of diabetes, and dementia caregiving, have been investigated [18,19,20]. Although CBR has many applications in the abovementioned clinical medicine training, medical education, disease diagnosis, and other fields, one study has pointed out that methods with high comprehensibility, such as CBR, may have relatively low accuracy [21]. Therefore, how to build and update the case base automatically, based on multisource and heterogeneous multimodal clinical case data, how to obtain the required knowledge quickly and accurately from a large amount of historical case data, and how to improve physicians’ adoption of a CBR knowledge system are problems that need to be solved urgently.

This paper introduces the case-based medical knowledge service system (CBR-MKS) on the basis of multimodal clinical big data and CBR to solve the above problems. CBR-MKS uses an information extraction method on the basis of clinical key feature information to intelligently establish a case base, quickly and automatically extract medical information, and obtain a high-quality case base through the evaluation mechanism of human-computer integration, which greatly improves the efficiency and automation of a medical case-base construction level. The CBR-MKS uses the weighted heterogeneous value distance measurement method (WHVDM) and the genetic algorithm of attribute weight learning for case matching through the interaction, feedback confirmation, and coordination of humans and machines to achieve accurate knowledge acquisition and enhance the interpretability, ease of use, and availability of knowledge recommendations. The following section introduces the application status, hotspots, and trends of the CBR method in the medical field, as well as the framework, key technologies, applications, and future research directions of the CBR knowledge service system.

## 2. Research Status of Medical CBR

Bibliometrics (the integrated use of mathematical and statistical knowledge) and a quantitative assessment of literature-related indicators are used to help reveal specific areas of the scientific knowledge panorama related to CBR [22,23]. This study uses the indexes SCI-E, SSCI, CPCI-S, ESCI, CCR-E, and IC in Web of Science (WOS) as data sources and uses advanced search methods to search #1 and #2, where #1 represents CBR and #2 represents electronic medical services. Specifically, #1 is TS = (‘case-based reasoning*’ or ‘case-based*’), while #2 is TS = (‘medical*’ or ‘health*’ or ‘hospital*’ or ‘disease*’). The literature type is ‘article,’ and a total of 2124 documents were collected.

### 2.1. Time Series Analysis of Volume of Articles

**Figure 1:**
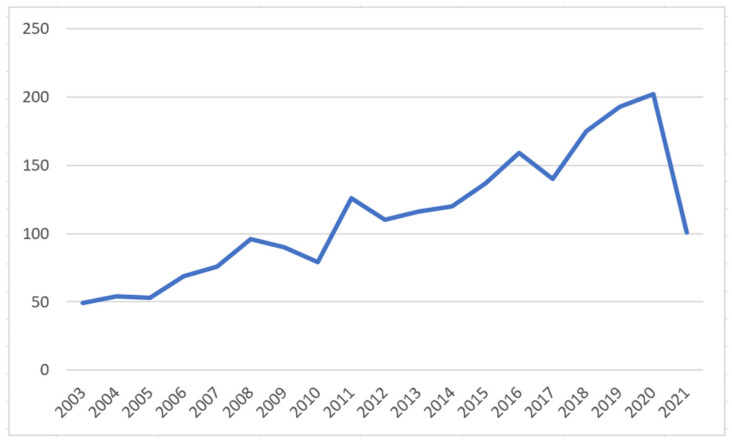
Number of CBR articles published in the medical and health fields over time.

Figure 1 shows the changes in the number of CBR articles published in the medical and health fields over time. From 2003 to 2005, research on CBR in the field of medicine was relatively stable, and the number of academic journal and conference articles published each year was about 50; from 2005 to 2008, the application of CBR in medical research increased, and the amount of literature published each year increased gradually; from 2008 to 2010, the research on CBR in medicine showed a short declining trend, but the decline was small; and from 2011 to 2020, the number of papers published fluctuated slightly, but the overall trend was one of rapid increase, reaching a peak in 2020.

### 2.2. Country and Region Distribution Analysis

**Figure 2:**
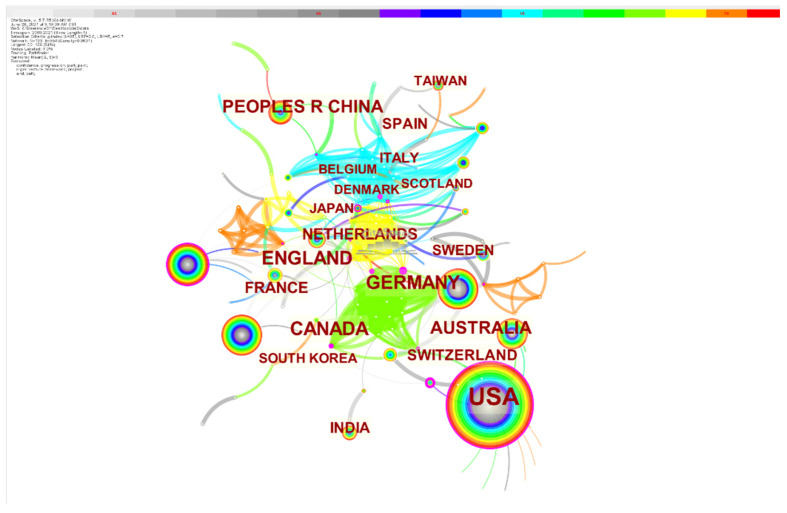
Top 20 countries and regions in terms of publication volume on CBR literature.

Among the top 20 countries or regions publishing CBR literature (Figure 2), 11 were in Europe, including England, Germany, the Netherlands, France, Switzerland, Spain, Italy, Sweden, Denmark, Scotland, and Belgium; five were in Asia, including China (the Chinese mainland), India, Taiwan (China), Japan, and Korea. In addition, the top 20 include the United States and Canada in North America, Brazil in South America, and Australia in Oceania. From the year of first publication in 2003, almost all of the top 20 countries and regions produced published in the following years. Only Denmark saw published CBR articles relatively late, starting in 2009. In terms of the number of publications, the United States had far more than the other countries and regions, accounting for 45.39% of all publications. Centrality indicates the importance of nodes. Among the top 20 countries with the largest number of publications, Brazil (0.22) had the highest centrality, followed by the United States (0.20) and England (0.13). Obviously, the United States, Canada, and Europe, represented by England and Germany, have great advantages in medical CBR research.

### 2.3. Institutional Distribution Analysis

A total of 525 institutions published articles on medical CBR, with an average of 4.15 articles per institution. The top 20 institutions issued a total of 498 articles, with an average of 24.9 articles per institution, which is much higher than the average number of all institutions. From the year of the first publication, the University of Toronto, University of California at San Francisco, University of Washington, WHO, Mayo Clinic, and other institutions show an earlier start in this field, while Harvard Medical College, Pittsburgh University, and Massachusetts General Hospital started later. The University of Toronto had the highest number and centrality of the top 20 institutions, which indicates obvious advantages in medical CBR research.

**Figure 3:**
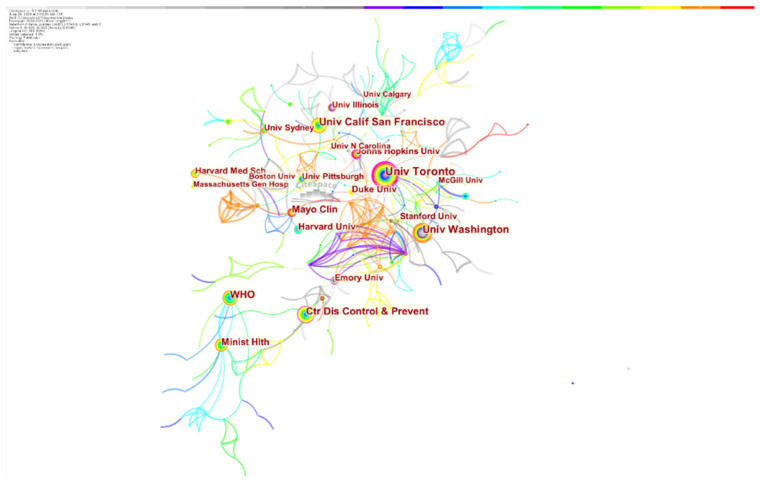
Institutional cooperation network related to CBR research.

As shown in Figure 3, the research institutions that produce medical CBR research have a close cooperative relationship. The University of Toronto, University of California at San Francisco, and University of Washington cooperate with many other institutions and are at the center of the cooperation network.

### 2.4. Analysis of Keyword Co-Occurrence

**Figure 4:**
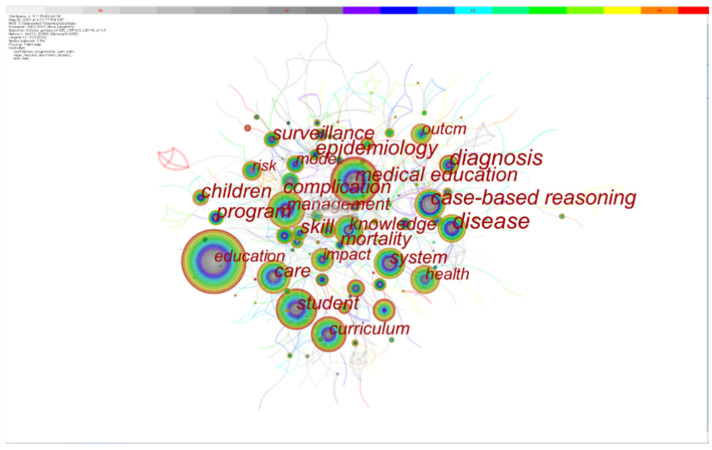
Keyword co-occurrence map.

Figure 4 shows the keyword co-occurrence network obtained by CiteSpace visualization analysis. Important keywords include CBR, disease, knowledge, children, technology, epidemiology, and surveillance. By studying the co-occurrence frequency and centrality of the keywords, it can be concluded that the research hotspots of medical case reasoning focus mainly on clinical medical training, nursing education, medical knowledge management, disease diagnosis, and other fields.

## 3. CBR-MKS System Framework Design

**Figure 5:**
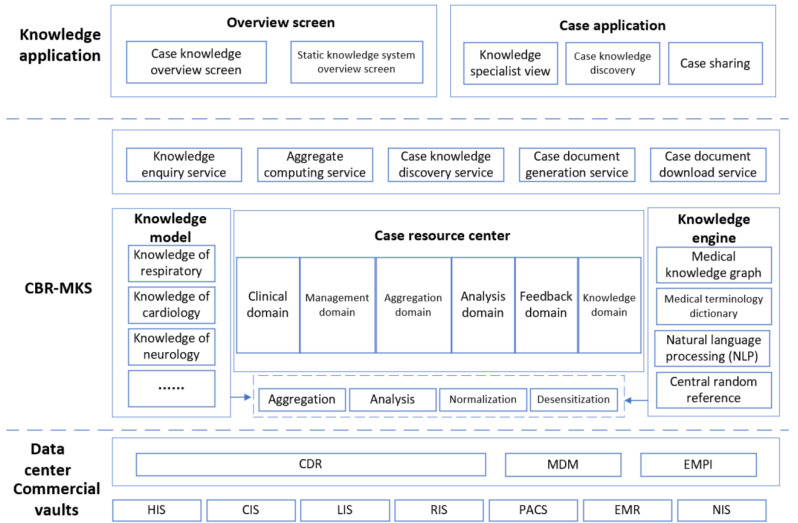
Overall framework of the CBR-MKS.

Based on an authoritative knowledge system, a summary of information from clinical experts, and big data on clinical health, CBR-MKS builds a general medical knowledge base, clinical case base, rare case base, and AI knowledge service engine (including case knowledge retrieval, recommendation, visual analysis, and other tools). CBR-MKS provides basic auxiliary diagnosis and treatment knowledge services, case knowledge services for clinical teaching, and full case knowledge services for clinical research for physicians in community health service centers, young physicians in hospitals, and physicians under regular training. Figure 5 shows the overall framework of the medical knowledge service system.

**Figure 6:**
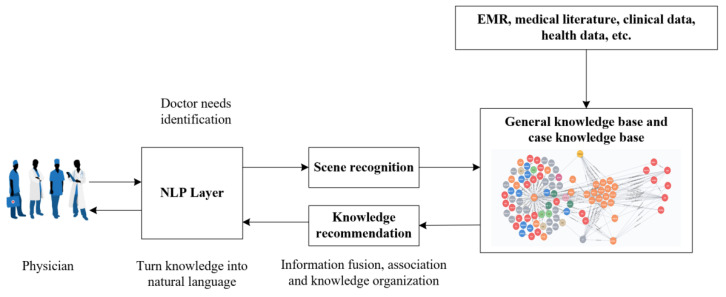
CBR-MKS knowledge recommendation process.

With the diagnosis of breast tumors by young physicians as an example, the knowledge recommendation process of the CBR-MKS is introduced, as shown in Figure 6. The CBR-MKS analyzes the examination and laboratory reports of patients with breast tumors, automatically extracts key feature information from medical big data, such as electronic medical and records to construct breast tumor case, and forms a breast cancer case base and rare case base. When a new patient comes to see a physician, the physician obtains the patient’s condition symptoms and basic examination information, and the system automatically matches similar cases on the basis of the patient’s symptoms and related basic examination information. Then, it recommends prediction results and complete cases (including diagnosis results, treatment plans, and prognosis) to the physician for decision-making support. In the CBR process, physicians could communicate with the system through natural language, put forward opinions and preferences for the recommendation scheme, or set weights for each feature attribute of the case in accordance with their own experience. The system could also rescreen, eliminate, combine, modify, reorder, and even re-recommend the case in accordance with the feedback information of the physician. The system generally recommends multiple historical similar cases for physicians’ reference to reduce the risk and possible harm that may be caused by recommending only the most similar case. Physicians need to make comprehensive judgments and decisions on the basis of the actual situation of the patient, their own experience, and their acquired knowledge. The process of knowledge recommendation in the CBR-MKS is a deep human-computer interaction and collaborative process. Physicians participate in the process of case knowledge acquisition rather than merely receiving the diagnosis results recommended by the system, thereby enhancing the interpretability and acceptability of the knowledge recommendations.

The CBR-MKS has strong adaptability and self-learning ability. The system can provide corresponding knowledge mining and recommend algorithm models according to different needs, and establish case correction rules and algorithms according to the guidance of physicians. According to the needs, the obtained case schemes are modified, optimized, and combined to adapt to the needs of knowledge schemes in various scenarios; the system has a complete set of case evaluation mechanisms. Only cases with a good evaluation by physicians can enter the case base. Through a reinforcement learning method, the system can automatically learn and form high-quality case evaluation rules, which provides the possibility for large-scale case quality evaluation. At the same time, with an increase in the number of high-level cases and medical institutions, the system completes the process of rapid knowledge accumulation and self-learning, and the reasoning ability of the system and the accuracy of knowledge discovery become higher and higher.

The CBR-MKS provides clinical cases and knowledge tools for clinical teaching in hospitals, clinical research, and assistance in diagnosis and treatment, all of which are conducive to the rapid growth of young physicians and reduce medical risks. In addition, we can make full use of the high-quality clinical case resources to extend the service ability of large hospitals, accelerate the distribution of high-quality medical resources to the community, and comprehensively improve the diagnosis and treatment ability of grass-roots physicians and the service level of community health service institutions.

## 4. Case Base Construction Method

**Figure 7:**
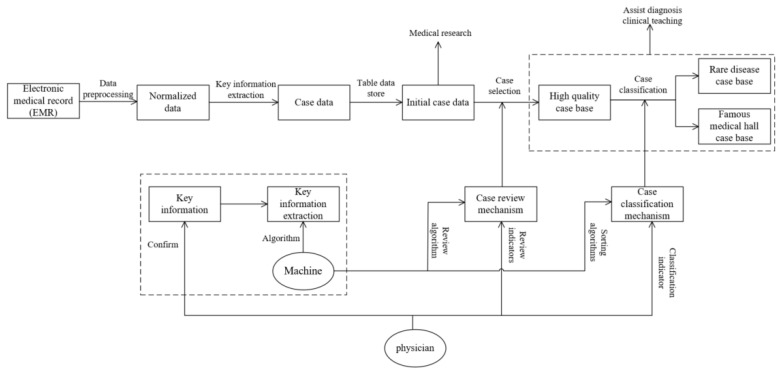
System data processing flow.

The CBR-MKS adopts a medical case knowledge base construction method based on clinical key feature information. In the construction process of this base, the physician’s clinical diagnosis reasoning process is fully integrated, and a collaboration between physicians’ professional knowledge and the machine algorithm is realized. The construction process conforms to the physician’s knowledge reasoning process and realizes the “interpretability” of case knowledge. The main process of case base construction is (1) data preprocessing, (2) key information extraction, (3) data storage, and (4) case selection, as shown in Figure 7.

1. Acquisition and pretreatment of electronic medical record data is used to obtain standardized data. The system acquires all kinds of medical record data from hospital information systems (HIS), laboratory information management systems (LIS), picture archiving and communication systems (PACS), and other software through its interface program and cleans the data in the report. The pretreatment process includes data removal, missing value processing, outlier processing, and so on.

2. According to the authoritative disease knowledge provided by physicians, the key feature information in the standardized data is extracted by a natural language processing method. This step extracts the key feature information from the standardized electronic medical record data and fully integrates the authoritative disease knowledge given by physicians in the extraction process, including the following:

i. According to the authoritative disease knowledge given by physicians (including clinical pathways, diagnostic guidelines, and disease consensus), the key feature information in the standardized data is determined; for example, with diabetes, this would include fasting blood glucose, postprandial blood glucose, glycosylated hemoglobin, and the oral glucose tolerance test.

ii. The structured numerical data and the corresponding index names in the standardized data are extracted by using the regular expression to obtain the key data information.

iii. The named entity recognition method is used to extract the unstructured text data from the standardized data and the corresponding entity reference, and the key text information is obtained. Specifically, this includes the following:

- According to the pre-trained medical word vector dictionary, the word vector matrix of the unstructured text data is obtained.

The existing research on the medical field’s word vector dictionary, trained based on the skip-gram training method, is adopted. The dimension of the medical field’s word vector dictionary is Z×d, the dimension of each word is d, the size of the dictionary is Z, and the input normalized data have the number of characters P. Afterward, embedded sentence $S=\left(c^{\left(1\right)}{,c}^{\left(2\right)},\cdots {,c}^{\left(p\right)},\cdots c^{\left(P\right)}\right)$, and the dimension becomes P×d.

- The word vector matrix is input into a plurality of pre-constructed classifiers to obtain the sentence sequence after word segmentation.

The pre-constructed weight matrix of multiple classifiers $\mathrm{WS}=\left({\mathrm{WS}}^{\left(1\right)}{,\;\mathrm{WS}}^{\left(2\right)},\;\cdots {,\;\mathrm{WS}}^{\left(i\right)},\;\cdots {\mathrm{WS}}^{\left(M\right)}\right)$, where ${\mathrm{WS}}^{\left(i\right)}$ is the weight of the *i*th classifier, and the value is between 0 and 1. The word vector matrix S is trained by multiple classifiers, and the output vector $H=\left(h^{\left(1\right)}{,h}^{\left(1\right)},\cdots {,h}^{\left(p\right)},\cdots {,h}^{\left(P\right)}\right)$, where $h^{\left(p\right)}$ represents the pth vector $\left(h^{\left(p\right)}\in R^{4\times M}\right)$, each row represents the probability that the character p belongs to the set {B: beginning character, M: middle character, E: end character, S: a single word}, and the columns represent different word splitters. Each column element in $h^{\left(p\right)}$ is multiplied by the weight ${\mathrm{WS}}^{\left(i\right)}$ of the corresponding classifier to get $h^{{\left(p\right)}^{\prime }}$, and then all the elements in each row of $h^{{\left(p\right)}^{\prime }}$, and then normalized according to the column to get the vector $h^{\left(c^{\left(p\right)}\right)}\left(h^{\left(c^{\left(p\right)}\right)}\in R^{4\times 1}\right){,\;\mathrm{where}\;h}^{\left(c^{\left(p\right)}\right)}=\left(h_{1}^{\left(c^{\left(p\right)}\right)}{,h}_{2}^{\left(c^{\left(p\right)}\right)}{,h}_{3}^{\left(c^{\left(p\right)}\right)}{,h}_{4}^{\left(c^{\left(p\right)}\right)}\right)$; $h^{\left(c^{\left(p\right)}\right)}$ is the line corresponding to the element with the largest value, which is the {B,M,E,S} label corresponding to the character, and the final word segmentation result is obtained.

- The sentence sequence after word segmentation is input into multiple pre-constructed part-of-speech markers to obtain the result of the part-of-speech markers.

If the word order is listed as $S^{\mathrm{ws}}=\left({\mathrm{ws}}^{\left(1\right)}{,\mathrm{ws}}^{\left(2\right)},\cdots {,\mathrm{ws}}^{\left(q\right)},\cdots {\mathrm{ws}}^{\left(Q\right)}\right)$, with $ws^{\left(q\right)}$ corresponding to the qth word, then ${\mathrm{ws}}^{\left(q\right)}=\left(h^{\left(c^{\left(1q\right)}\right)}{,h}^{\left(c^{\left(2q\right)}\right)},\cdots h^{\left(c^{\left(\mathrm{tq}\right)}\right)}{,h}^{\left(c^{\left(\mathrm{Tq}\right)}\right)}\right)$; with ${\mathrm{ws}}^{\left(q\right)}$ corresponding to the number of characters T, then ${\mathrm{ws}}^{\left(q\right)}$ corresponds to each character vector $h^{\left(c^{\left(\mathrm{tp}\right)}\right)}\left(h^{\left(c^{\left(\mathrm{tp}\right)}\right)}\in R^{4\times 1}\right)$. The column vector $w^{q}\;\left(w^{q}\in R^{4\times 1}\right)$ of each word is obtained by adding each row element of ${\mathrm{ws}}^{\left(q\right)}$ and normalizing it by column. The vector of the segmentation sequence $S^{\mathrm{ws}}$ is expressed as $S^{V}=\left(w^{\left(1\right)}{,w}^{\left(2\right)},\cdots {,w}^{\left(q\right)},\cdots w^{\left(Q\right)}\right)$.

$S^{V}$ is input into multiple part-of-speech markers for training, and the weight of the marker is ${\mathrm{WT}=(\mathrm{WT}}^{\left(1\right)}{,\mathrm{WT}}^{\left(2\right)},\cdots {,\mathrm{WT}}^{\left(j\right)},\cdots {\mathrm{WT}}^{\left(N\right)})$, with ${\mathrm{WT}}^{\left(j\right)}$ for the first *j*, a marker weight, and the value is between 0 and 1. After each multiple marker training, the output vector $E=\left(e^{\left(1\right)}{,e}^{\left(1\right)},\cdots {,e}^{\left(q\right)},\cdots {,e}^{\left(Q\right)}\right)$.

$e^{\left(q\right)}$ represents the qth character vector ($e^{\left(q\right)}\in R^{7\times N}$), and each row represents the probability that the word q is

{CL1:“patient information,” CL2:“time,” CL3:“disease,” CL4:“symptom,” CL5:“examination test,” CL6:“treatment plan,” CL7:“other”}

and the column represents the vector corresponding to different classifiers. The elements in each column of $e^{\left(q\right)}$ are multiplied by the weight ${\mathrm{WT}}^{\left(j\right)}$ of the corresponding classifier to obtain $e^{{\left(q\right)}^{\prime }}$, and then all the elements in each row of $e^{{\left(q\right)}^{\prime }}$ are added. After normalization by column, the corresponding row of the elements with the largest median value of the vector $e^{\left(w^{\left(q\right)}\right)}\left(e^{\left(w^{\left(q\right)}\right)}\in R^{7\times 1}\right)$, $e^{\left(w^{\left(q\right)}\right)}$ is the category label of {CL1, CL2, CL3, CL4, CL5, CL6, CL7} corresponding to the character, and the final part-of-speech marking results are obtained.

- According to the part-of-speech tagging results, the key text information is obtained.

The first loss function in the pre-constructed classifier training process is as follows:

(1) $$\mathrm{Loss}1=\frac{1}{P}\overset{P}{\underset{i=1}{\sum }}\left({1-h}_{\mathrm{true}}^{\left(c^{\left(p\right)}\right)}\right)$$

where $h_{\mathrm{true}}^{\left(c^{\left(p\right)}\right)}$ is the corresponding probability value of the correct character label, $h_{\mathrm{true}}^{\left(c^{\left(p\right)}\right)}\in \left[0,1\right]$; *P* represents the total number of characters; and *p* represents the *p*th character.

The second loss function in the pre-constructed part-of-speech marker training process is as follows:

(2) $$\mathrm{Loss}2=\frac{1}{Q}\overset{Q}{\underset{i=1}{\sum }}\left({1-e}_{\mathrm{true}}^{\left(w^{\left(q\right)}\right)}\right)$$

where $e_{\mathrm{true}}^{\left(w^{\left(q\right)}\right)}$ is the corresponding probability value of the correct character label, $e_{\mathrm{true}}^{\left(w^{\left(q\right)}\right)}\in \left[0,1\right]$; *Q* represents the total number of characters; and *q* represents the *q*th character.

The calculation of the total loss function is as follows:

(3) Loss=Loss1+Loss2

The overall loss function is minimized to update the weights of the multiple classifiers and multiple markers.

iv. The key data information and key text information are matched according to the patient ID, and the key feature information is obtained.

- According to the key feature information, the initial case knowledge base is obtained.
- According to the audit index proposed by physicians for disease knowledge, high-quality cases are selected from the initial case knowledge base, and the high-quality case knowledge base is obtained.

According to the audit index $X=\left(x_{1}{,x}_{2},\cdots {,x}_{i},\cdots \right)$, where $x_{1}$ represents knowledge richness, $x_{2}$ represents curative effect or treatment time, and $x_{n}$ represents other audit index sub-items, the named entity recognition method is used to extract the key information of case evaluation in the initial case knowledge base, and the case score Score1 is calculated.

(4) $$\mathrm{Score}1=\mathrm{sigmoid}\left(a_{1}x_{1}{+a}_{2}x_{2}{+a}_{3}x_{3}+\cdots {+a}_{n}x_{n}\right)$$

(5) $$\mathrm{sigmoid}\left(x\right)=\frac{1}{{1+e}^{-x}}$$

where $a_{1}{,\;a}_{2}{\ldots \ldots a}_{n}$ represents the weight; formula (5) represents the normalization function.

When Score1≥σ (0≤σ≤100), the corresponding case is marked, and high-quality cases are screened to obtain the high-quality case knowledge base. The σ represents the first target score threshold, which can be set as required.

- According to the classification indices proposed by physicians for ease of understanding and rare cases, the high-quality case knowledge base is classified, and the case knowledge bases of cases by expert physicians and rare diseases are obtained.

According to the case classification index $Z=\left(z_{1}{,z}_{2},\cdots {,z}_{i},\cdots \right)$ proposed by the well-known physicians for the ease of understanding of the case, where $z_{1}$ denotes the accuracy of the wording of the case, $z_{2}$ denotes the simplicity of the case, and $z_{i}$ denotes other sub-items of the case classification index of the well-known physician, syntactic analysis methods are used, such as Lexical Analysis of Chinese (LAC) tools, to obtain the case classification index information of the well-known physician from the high-quality case knowledge base and calculate the case score Score2:

(6) $$\mathrm{Score}2=\mathrm{sigmoid}\left(b_{1}z_{1}{+b}_{2}z_{2}{+b}_{3}z_{3}+\cdots {+b}_{n}z_{n}\right)$$

where $b_{1}{,\;b}_{2}{\ldots \ldots b}_{n}$ represents the weight.

When Score1≥γ (0≤γ≤100), the corresponding cases are marked and selected to obtain the case knowledge base of the well-known hospital. The γ represents the second target score threshold, which can be set as required.

According to the classification index of rare diseases proposed by physicians for rare degrees, the classification index of rare diseases includes the frequency of disease occurrence Y, and the frequency of disease occurrence ω is counted by statistical methods. When the frequency of disease occurrence Y is lower than *ω* (0<ω<1), where ω represents the third target score threshold, which can be set according to needs, the case is marked as a rare disease case. After classifying the high-quality case knowledge base, the rare disease case knowledge base is obtained.

In hard equipment, when key feature information is extracted with a string length of less than 100, approximately 1400 cases could be identified per minute on average, and 530 cases could be stored by feature transformation per minute under the single-threaded condition. The efficiency could vary with the specific memory conversion efficiency and disk I/O limit. The model realizes the post-structuring of the text and automatically recognizes the characteristic entities appearing in the medical text.

## 5. Case Knowledge Matching Model

The selective weighted heterogeneous value distance measure (WHVDM) was adopted, which improved the traditional Euclidean distance measure and value difference measure (VDM), and made both continuous and discrete variables applicable [18]. The specific case matching algorithm is as follows.

The case is described as a set of (*x*, *y*) vectors, where $x=\left(x_{1}{,x}_{2}{,\ldots ,x}_{n}\right)$ is a vector of independent variables of characteristic attributes and *y* ∈ *Y*; *Y* is a discrete variable of the corresponding class. A class value in a case base that stores a set of solved historical cases is known. Given a new unresolved target case, where the class value is unknown, CBR aims to retrieve a group of cases that are considered to be most similar to the new case from the case base and to support decision makers to predict the class value. The relative importance of an attribute measuring the distance between a new target case and a stored case is reflected in the weight of a vector $w=\left(w_{1}{,w}_{2}{,\ldots ,w}_{n}\right)$, where,

$$0\leq w_{i}\leq 1\;i=1,2,\ldots ,n\;,\;\overset{n}{\underset{i=1}{\sum }}w_{i}=1$$

The WHVDM between new target case *t* and storage case *r* is defined as follows:

$$\mathrm{WHVDM}\left(t,r\right)=\sqrt{\sum_{i=1}^{n}w_{i}d_{i}^{2}\left(t,r\right)}$$

where,

(7) $$d_{i}^{2}\left(t,r\right)=\{\begin{array}{ll} \mathrm{vdm}\left(t,r\right), & \mathrm{if}\;x_{i}\;\mathrm{is}\;\mathrm{discrete}\\ {\mathrm{diff}}^{2}\left(x_{t,i},x_{r,i}\right),\; & \mathrm{if}\;x_{i}\;\mathrm{is}\;\mathrm{continuous} \end{array}$$

In formula (7), vdm(t,r) is a VDM proposed by Stanfill and Waltz [24]. The VDM between the discrete attribute $x_{i}$ of the target case *t* and the storage case *r* is defined as follows:

(8) $$\begin{array}{c} {\mathrm{vdm}}_{i}\left(t,r\right)=\sum_{a\in Y}\left({\mathrm{Pr}(y=a|x}_{i}{=x}_{t,i}\right){-\mathrm{Pr}(y=a|x}_{i}{=x}_{r,i}{))}^{2}\\ •\sqrt{\sum_{a\in Y}{\mathrm{Pr}(y=a|x}_{i}{=x}_{t,i}{)}^{2}} \end{array}$$

In formula (7), ${\mathrm{diff}}^{2}\left(x_{t,i}{,x}_{r,i}\right)$ is a part of a Euclidean distance measurement in various CBR systems [25].

Specifically, given new target case *t* and storage case *r*,

(9) $${\mathrm{diff}}^{2}\left(x_{t,i}{,x}_{r,i}\right){=(x}_{t,i}{-x}_{r,i}{)}^{2}$$

Genetic algorithm (GA) was used to learn attribute weights on the basis of sample cases. Given a set of reference cases R and a set of test cases T, the most similar reference case is used to predict the category value of each test case, and the number of test cases that are correct is calculated. $s(t)=\underset{r\in R}{\mathrm{argmin}}\mathrm{WHVDM}\left(t,r\right)$ denotes the reference case most similar to the test case. The number of correctly predicted test cases is $\sum_{t\in T}{I(y}_{t}{=y}_{s(t)})$, where *I* is the indicator function, namely $I(e)=\{\begin{array}{l} 1,\;\mathrm{if}\;e\\ 0,\;\mathrm{otherwise} \end{array}$.

The case matching model was verified using two different stages of studies [25]. In the first stage, WHVDM-GA was compared with other CBR approaches on the basis of the Euclidean distance algorithm, WE-Expert, GCBR-IE, WHVDM-Expert, and WHVDM-IE. The accuracy of WHVDM-GA was improved by at least 3.6% and 4.3% by the two studies on the basis of different datasets. The F-value improved by at least 4.6% and 4.0%. In the second stage, the method was further compared with RBF Network, CART, Logistic Regression, and Naive Bayes, which are all commonly used knowledge discovery methods. The accuracy improved by at least 3.2% and 8.9% on the basis of the two similar datasets. Correspondingly, the F-value increased by at least 4.5% and 9.2%.

## 6. Pilot Application Evaluation

We invited fifteen young physicians (under the age of 35) in urban hospitals and in grass-roots hospitals who had used a CBR-MKS and a clinical decision support system (CDSS), respectively, to score the two systems. The score was based on the ten dimensions of knowledge adoption, ease of use, physician participation, usefulness of improving medical quality, satisfaction of system use, interpretability of recommendation scheme, perceived security, ability enhancement, continuance intention, and recommendation intention.

We used the nonparametric measurement method Kendall’s W to verify the consistency of different physician evaluations. The Kendall synergy coefficient is a nonparametric test method for multiple paired samples that can be used to analyze whether the judge’s criteria are consistent. The Kendall synergy coefficient is between 0 and 1, and the closer to 1, the stronger the consistency. In this study, Kendall’s W is 0.845, indicating that the evaluators’ opinions are highly consistent. Therefore, the evaluation results of the fifteen physicians were taken as the average, as shown in Table 1.

The results showed that both grass-roots physicians and young physicians had a high degree of acceptance of auxiliary medical equipment and considered its results to be satisfactory. However, the score of the auxiliary medical system based on CBR was higher than that of the CDSS auxiliary medical system on the whole and at a high level, with an average of more than 8.5 points. The CBR-MKS is particularly prominent in knowledge adoption, participation, and interpretability, which is due to its good human-computer interaction coordination. In contrast with the CDSS, which directly gives diagnosis recommendations, the CBR-MKS can match similar cases, which is convenient for physicians to analyze and diagnose, and makes the diagnosis results more evidence-based. In the process of diagnosis and treatment, the self-adaptability and self-learning ability of the CBR-MKS make the cases recommended by the system through knowledge accumulation and learning more and more accurate. Based on the above advantages, physicians are more willing to continue to use the CBR-MKS and are willing to recommend it to their peers. In addition, the hospital evaluated and fed back effectiveness of the CBR system use and found that physicians’ average adoption rate of CBR-MKS was approximately 19% higher than that of CDSS, and physicians’ average diagnosis accuracy improved 5.1% compared with that before the system was introduced. Further, our empirical study showed that the CBR-MKS is beneficial to the improvement of organizational performance [26].

## 7. Limitations and Future Works

Although the CBR-MKS has been proven to be beneficial to auxiliary diagnosis and treatment, the limitations and potential risks caused by its use still need to be considered.

1. Although the accuracy of the system’s recommendation algorithm is already high, it is still not 100%, and the system still has the possibility of recommending error cases. Under this circumstance, if the physician could not conduct comprehensive research and judgment on the basis of the actual condition of the patient combined with his or her own experience and apply the results recommended by the system rigidly, it may bring more serious consequences. Before using the system, physicians need to be trained on the operating specifications, risks, and ethics to help them use the system correctly and clarify the potential risks that may exist in the system.
2. A large number of historical cases contain private information, such as the patient’s name, disease, and contact information. A risk of patient privacy disclosure is present in the process of opening and sharing case data from one medical institution to another. Although some patient privacy information (including name, contact information, ID card number, and other sensitive privacy information) has been masked or removed to ensure patients’ privacy during CBR-MKS use, and different users are set different access and browsing permissions for information, a stricter patient privacy protection mechanism needs to be constructed in the system to further reduce the risk of disclosure of patient privacy information due to high sensitivity of medical data.

## 8. Conclusions

The study found that CBR-MKS uses an information extraction method based on clinical key feature information to intelligently establish a case base and uses WHVDM and attribute weight learning using GA for case matching, which can greatly improve the efficiency of case base construction and is easier to understand, trust, and adopt by physicians than other auxiliary diagnosis and treatment methods. It provides decision-making reference for young physicians and grass-roots ones, improves diagnosis and treatment level, and enhances implementation effectiveness of the hierarchical medical treatment system.

## Figures and Tables

**Table 1:** Average evaluation results of CBR-MKS and CDSS.

Assessment Item of CBR-MKS	Degree of Approval (10 Full Points)	Assessment Item of CDSS	Degree of Approval (10 Full Points)
1. Whether the system promotes knowledge adoption	9.11	1. Whether the system promotes knowledge adoption	6.89
2. Whether the system is easy to use	8.77	2. Whether the system is easy to use	7.55
3. Whether the system facilitates your participation	8.73	3. Whether the system facilitates your participation	4.93
4. Whether the system is useful for improving the quality of care	8.65	4. Whether the system is useful for improving the quality of care	6.92
5. Whether the system meets your expectations	8.85	5. Whether the system meets your expectations	6.07
6. Whether the system recommended solution can be interpreted	9.01	6. Whether the system recommended solution can be interpreted	4.48
7. Whether the system is considered reliable	9.09	7. Whether the system is considered reliable	8.94
8. Whether the system helps to improve your capabilities	9.22	8. Whether the system helps to improve your capabilities	7.72
9. Are you willing to continue using the system?	9.34	9. Are you willing to continue using the system?	6.95
10. Are you willing to recommend the system to peers for use?	8.89	10. Are you willing to recommend the system to peers for use?	5.43

## Data Availability

The data presented in this study are available on request from the corresponding author. The data are not publicly available due to the request of respondents.
